# Endothelial Cell Adhesion Molecules- (un)Attainable Targets for Nanomedicines

**DOI:** 10.3389/fmedt.2022.846065

**Published:** 2022-04-07

**Authors:** Nenad Milošević, Marie Rütter, Ayelet David

**Affiliations:** Department of Clinical Biochemistry and Pharmacology, Faculty of Health Sciences, Ben-Gurion University of the Negev, Beer-Sheva, Israel

**Keywords:** cancer, inflammation, active drug targeting, diagnosis, vascular endothelial cells, imaging

## Abstract

Endothelial cell adhesion molecules have long been proposed as promising targets in many pathologies. Despite promising preclinical data, several efforts to develop small molecule inhibitors or monoclonal antibodies (mAbs) against cell adhesion molecules (CAMs) ended in clinical-stage failure. In parallel, many well-validated approaches for targeting CAMs with nanomedicine (NM) were reported over the years. A wide range of potential applications has been demonstrated in various preclinical studies, from drug delivery to the tumor vasculature, imaging of the inflamed endothelium, or blocking immune cells infiltration. However, no NM drug candidate emerged further into clinical development. In this review, we will summarize the most advanced examples of CAM-targeted NMs and juxtapose them with known traditional drugs against CAMs, in an attempt to identify important translational hurdles. Most importantly, we will summarize the proposed strategies to enhance endothelial CAM targeting by NMs, in an attempt to offer a catalog of tools for further development.

## Introduction

Recent failures of advanced nanomedicine (NM) drug candidates (due to lack of efficacy or dose limiting toxicity) (1) inspire a drop in the nanomedicine “hype” (2, 3). Among the reasons for modest success of clinical translation of NM are the poor accumulation of injected nanoparticles at the site of inflammation and solid tumor and the poor tissue penetration of NM (4, 5). One of the proposed strategies to overcome these limitations is to actively direct NM toward the disease-related vasculature via endothelial cell adhesion molecules (eCAMs-for the sake of simplicity we will use the abbreviation CAM) using high-affinity binding ligands. Many of these targets exhibit their function on the endothelium of blood vessels in the vicinity of inflammatory processes and are mainly absent from the non-activated endothelium (6, 7). Furthermore, targeting CAMs eliminates the need for deep tissue penetration, as these targets are directly accessible for NMs in the blood stream.

Structurally, CAMs like selectins (E-, P- and L-selectin), vascular cell adhesion molecule 1 (VCAM-1), intracellular adhesion molecule 1 (ICAM-1), and integrins are transmembrane glycoproteins, and their expression is correlated with the presence of inflammatory signaling via cytokines and chemokines in the proximity of tissue damage. Their main function is to facilitate the process of leukocyte infiltration from the blood stream into the inflamed tissues, by forming bonds with ligands on these circulating cells, allowing for rolling on the endothelium, adhesion and trans-endothelial migration (8, 9). This role makes CAMs crucial in acute response to tissue damage and acute inflammation, however in chronic inflammation excessive or prolonged infiltration of leukocytes can lead to further damage. Binding of NMs to CAMs can improve the vascular uptake and therapeutic efficacy of drug molecules entrapped within, or attached to the nanomedicine formulation. Due to their distinct expression patterns linked to the inflammatory state of the proximate tissue, CAMs are viable disease and progression markers (10, 11), hence NMs targeting CAMs depict a useful tool for vascular imaging (12). Furthermore, successful binding of CAMs can impair their function as anchors supporting leukocyte infiltration, translating to a reduction of inflammation severity. Lastly, presence of CAM ligands on CAR T-cells was shown to improve their homing to tumors or in case of leukemias, to the bone marrow (13). Glycoengineering of the CAR T-cell surface receptors could promote E-selectin binding for improved bone marrow or any inflammatory site homing (14). For these reasons, CAMs are recognized as viable targets for controlling inflammation, especially in the field of NM.

In the present manuscript, we highlight the key studies in which endothelial CAM-targeted NM formulations were explored to improve drug delivery, to enhance vascular imaging or to disrupt CAM-mediated activity in pre-clinical settings, and we discuss how these approaches can be employed to enhance clinical translation. This mini-review will focus on the CAMs that are considered as early markers of endothelial activation and dysfunction, e.g., selectins (E-, P-and L-selectin), ICAM-1 and VCAM-1. Integrins, which are expressed on endothelial cells, but they are also present in a variety of tumor cells, are discussed as a separate group of vascular adhesion molecules.

## Physiological Role of CAMs

**Scheme 1:**
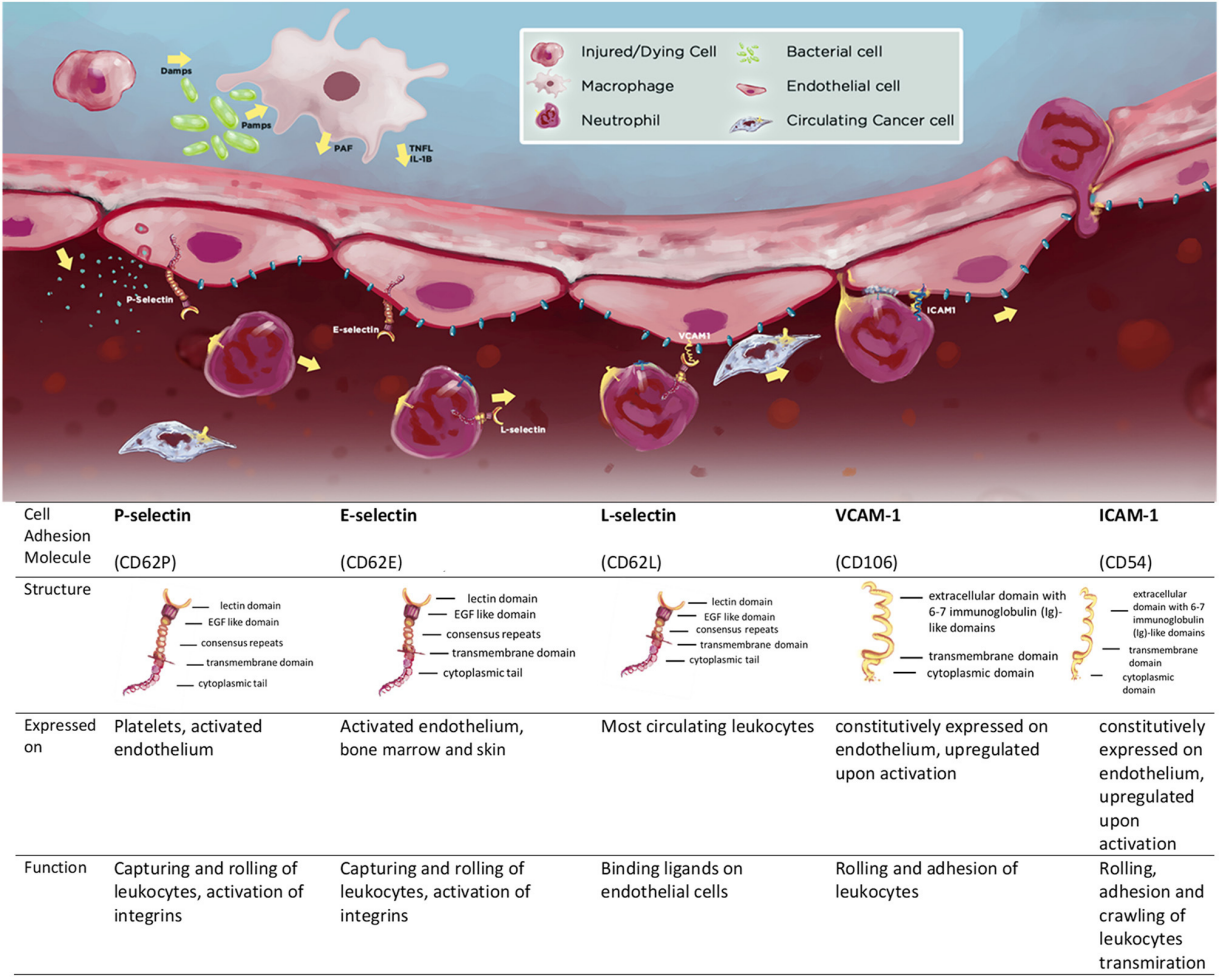
Cell adhesion molecules (CAMs) P-, E-, and L-selectin, VCAM-1, ICAM-1 and the summary of their structure and function; expression patterns and function.

The major endothelial expressed CAMs can be divided into integrins, selectins (E- and P-selectin), and immunoglobulin superfamily members (ICAM-1, VCAM-1). L-selectin is expressed on the membranes of immune cells (7). The structure of these transmembrane glycoproteins is discussed in detail elsewhere (6, 7) and summarized in Scheme 1.

The main functions of CAMs become apparent following “endothelial activation”- a process of profound changes on the vascular endothelium induced by inflammatory mediators (9). Resident immune cells (such as tissue-resident macrophages) act as “first responders” by releasing inflammatory cytokines that activate endothelial cells and release chemokines to recruit circulating inflammatory cells (8, 9). Once activated, endothelial cells can also produce cytokines and chemokines, stimulating further migration and accumulation of leukocytes in the affected tissues (9). Under non-inflammatory conditions, circulating leukocytes would not adhere to the endothelium, however, upon activation endothelial cells start to express CAMs. In a matter of minutes, as a rapid response to mediators such as histamine or platelet-activating factor (PAF), pre-synthesized CAMs (e.g., P-selectin) are transported to the surface of cells. Other mediators, such as cytokines (TNFα, IL-1β) induce a more gradual activation of endothelial cells including transcriptional induction and synthesis of numerous other CAMs over hours (15). In addition, leukocytes themselves become activated and change the expression patterns of CAMs on their surface (α_4_β_1_ also known as very late antigen-4 or VLA-4, and lymphocyte function-associated antigen-1, LFA-1), making them more adhesive to endothelial cells. Together these changes facilitate a multistep process of leukocyte transendothelial migration (Scheme 1). In a defined sequence of events, leukocytes initially form transient connections with selectins on endothelial cells, causing them to slow down and start to roll on their surface. Conformational changes in leukocyte-expressed integrins induce the formation of high-affinity bonds through binding to other CAMs (VCAM-1, ICAM-1) (16). In addition, vasoactive substances from the activated endothelium increase the diameter of the blood vessels, reducing intravascular pressure and increasing blood flow, allowing higher chances for circulating leukocytes to form bonds with CAMs successfully. In the final stage, leukocytes transmigrate through the endothelial cell layer, basement membrane, and pericytes, and reach the tissue. Once there, these leukocytes collaborate with tissue-resident macrophages to exert their effector functions (17). Usually, after the trigger for the inflammation is eliminated, the inflammatory process is directed to resolution. This crucial step can be absent or altered in several chronic immune-mediated inflammatory diseases (IMID e.g., rheumatoid arthritis, psoriasis, and Crohn's disease), where CAMs play a critical role (18). They stay up-regulated in the inflamed tissues, over a prolonged period of time, while the extent of their presence is linked to the severity of the disease. The excessive recruitment of leukocytes by CAMs can propagate the inflammatory process and, in many cases, aggravate tissue injury (18).

## Pathological Conditions With CAM Implications

Leukocyte adhesion mediated by CAMs is crucial in (patho)physiological events such as acute inflammation, immune response, wound repair, and hemostasis (7, 8, 15, 19). More recently, their role in chronic inflammation, promoting cancer metastases, and other pathological processes has been described in more detail (20, 21). This led researchers to emphasize the ambiguity of CAMs, for example, selectins, as having the nature of Dr. Jekyll and Mr. Hyde (11). The “good” face represents the physiological role of selectins, while the “evil” one becomes dominant whenever the leukocyte adhesion is dysregulated, as during chronic inflammation. This imbalance is well described in IMIDs such as rheumatoid arthritis, Chron's disease, and in other chronic cardiovascular or neurological diseases (18, 22).

The implications of CAMs in pathologies were extensively studied and confirmed on several levels. In some cases, CAMs show elevated plasma levels of their soluble forms in patients suffering from chronic diseases: soluble E-selectin in inflammatory bowel disease, bronchial asthma, atopic dermatitis (10) as well as in hypertension, diabetes, and hyperlipidemia (23); soluble P-selectin in cardiovascular diseases, especially in deep vein thrombosis (24). Not only are soluble VCAM-1 and ICAM-1 elevated in the plasma of breast cancer patients (25), but higher VCAM-1 serum levels in patients with epithelial ovarian cancer were also linked with disease progression and metastasis (26). There is evidence that elevated levels of E-selectin and ICAM-1 may also serve as molecular markers for atherosclerosis and the development of clinical coronary heart disease (27, 28).

Secondly, tissue up-regulation of CAMs was confirmed in clinical samples taken from patients suffering from various inflammatory diseases. Elevated levels of E-selectin were detected in liver sections of patients with alcoholic liver disease, bronchial biopsies in chronic bronchitis, and synovium in rheumatoid arthritis (10). Patients with sickle cell disease (SCD) express higher levels of P-selectin, which is thought to contribute to the adhesion of sickle red blood cells to the endothelium (29, 30). Higher VCAM-1 expression was, for example, associated with higher eosinophil infiltration into the inflamed lungs of asthmatic patients, rejection of liver allografts, the severity of RA, and cancer progression (31).

Furthermore, animal disease models are providing valuable insight into CAM involvement in pathological processes. E-selectin was identified as a target in rodent models of asthma, myocardial infarction, stroke, and inflammatory bowel disease (IBD) (10, 32). VCAM-1 was upregulated in a model of experimental allergic encephalomyelitis (33) and aortic sections in collagen-induced murine model or rheumatoid arthritis (34).

Finally, drugs that exert anti-inflammatory effects, especially on the level of the vasculature, decrease the levels of CAMs and leukocyte adhesion. Statins reduce VCAM-1 protein levels and E-selectin mRNA levels *in vitro* in TNFα activated HUVECs (35, 36), as confirmed in hypercholesterolemic patients (37). Therapy with methotrexate (MTX) reduced expression levels of E-selectin and VCAM-1 in synovial tissues of patients with rheumatoid arthritis (38). Similarly, some natural products, like herbal of fungal extracts, can reduce expression of CAMs on activated endothelial cells (39, 40). Also, interventions that influence vascular health and reduce endothelial inflammation, like smoking cessation (sICAM-1) (41) and physical exercise (E-selectin, ICAM-1) (42), have been shown to reduce CAM plasma levels.

Together, these findings indicate that CAMs are viable pharmacological targets in conditions where leukocyte infiltration is dysregulated.

## Drug Candidates-Inhibitors of CAMs: mAbs and Small Molecules

Successful examples of clinically approved inhibitors of CAMs are limited to three monoclonal antibodies (mAbs): natalizumab and vedolizumab, which target leukocyte expressed α_4_β_7_ integrins and are approved for the treatment of IBD (43) and crizanlizumab (anti-P-selectin mAb)-approved in 2019 for vaso-occlusive crisis (VOC) in SCD (29). Table 1 summarizes some examples of CAMs inhibitors that were tested in clinical trials (CTs) and their implications in human diseases. Another mAb against P-selectin, inclacumab, is currently being developed for therapy of VOC in SCD (44). Aselizumab (anti-L-selectin mAb) was developed for controlling the infiltration of activated neutrophils into different inflamed organs. It was expected that targeting L-selectin, expressed on all leukocytes, would lead to better systemic effects than blocking regionally expressed E- or P-selectin. Clinical trial results showed a lack of efficacy, even though the saturation of leukocyte L-selectin was 89%. Possible explanations could be that the remaining 10% of L-selectin could still be sufficient to mediate adhesion or that other CAMs could compensate for L-selectin blockage (45, 46). The murine anti-ICAM-1 mAb, enlimomab, reached clinical-stage development for stroke treatment, following the rationale that ICAM-1 blockade inhibits reperfusion-induced inflammation with neuronal injury after stroke. Even though preclinical studies demonstrated a reduction in the extent of reperfusion injury, clinical results showed negative effects on patient survival and symptom-free recovery (47). Besides mAbs, another biological CAM inhibitor was tested. Recombinant P-selectin glycoprotein ligand IgG fusion protein (YSPSL) failed in CTs for protecting allograft function after transplantation, which stopped further development (48, 49).

**Table 1:** Cell adhesion molecules (CAMs) P-, E-, and L-selectin, VCAM-1, ICAM-1 and their implications in human diseases; natural ligands, and low Mw inhibitors/monoclonal antibody drug candidates.

**CAM**	**P-selectin**	**E-selectin**	**L-selectin**	**VCAM-1**	**ICAM-1**
Implication in human diseases	CVS diseases, sickle cell disease (vaso-occlusive crisis), different types of cancer	CVS disease, rheumatoid arthritis, COPD and asthma, psoriasis, vaso-occlusive crisis in SCD, cancer	Sepsis, multiple organ failure	CVS disease, rheumatoid arthritis, asthma, transplant rejection, and cancer	Atherosclerosis and other CVS diseases, Breast cancer
Natural ligands	PSGL-1 sulfated polysaccharides (heparin)	Structures with sLe^X^ and sLe^A^ ESL-1 PSGL-1 CD44 Enforced hematopoietic cell E- and L-selectin ligand (HCELL)	PSGL-1 sulfated polysaccharides E-selectin Enforced hematopoietic cell E- and L-selectin ligand (HCELL)	VLA-4 (integrin α4β1) MAC1	Lymphocyte function-associated antigen 1 (LFA1, or αLβ2 integrin)
Small molecule inhibitor	Bimosiamose (pan-selectin inhibitor) Sevuparin-failed in CTs for Sickle Cell disease	Cylexin (CY-1053) Rivipansel (GMI-1070) Uproleselan (s.c. version- GMI-1687) Efomycine M-failed in CTs Bimosiamose (pan-selectin inhibitor)	Sevuparin-failed in CTs for SCD Bimosiamose (pan-selectin inhibitor)	/	/
Monoclonal antibodies and recombinant proteins	Crizanlizumab-approved (FDA Nov 2019) for VOC in SCD Inclacumab-in CTs for SCD diseases, after failing in CTs for CVS diseases YSPSL (rPSGL-Ig) -renal allograft-failed in clinical trials	/	Aselizumab-failed in CTs for severely injured patients	/	Enlimomab-failed in CTs, acute stroke, kidney allograft and multiple myeloma

*CTs, clinical trials; COPD, chronic obstructive pulmonary disease; CVS, cardiovascular disease; SCD, sickle cell disease; VOC, vaso-occlusive crisis*.

Apart from monoclonal Abs, many attempts were made to develop small molecule inhibitors of CAMs. An array of natural products isolated from plant material (terpenoids, lignans etc.), or from microbial (efomycine M) or marine origin, have initially demonstrated *in vitro* inhibition of CAMs, however, no further clinical development can be identified (50). Employing the rational design-driven approach, a series of glycomimetic inhibitors were derived from the structure of the selectin ligand sialyl Lewis X (sLe^X^). One of the first analogs of sLe^X^, pentasaccharide cylexin (CY-1053), was discontinued due to poor efficacy in CTs for reperfusion injury (51), likely due to the low metabolic stability and rapid clearance associated with the carbohydrate structure (11). “Second-generation” pan-selectin inhibitor bimosiamose (binding to E-selectin, P-selectin, and L-selectin) has minimal carbohydrate structural motives and was developed for the treatment of psoriasis and airway inflammation (52–55). Even though inhalation of bimosiamose induced a reduction of airway inflammation in chronic obstructive pulmonary disease (COPD) patients, it appears that it was discontinued (49, 56). Rivipansel (GMI-1070), another pan-selectin inhibitor (more active on E-selectin), is being developed for the treatment of SCD. In a phase III CT, rivipansel did not meet the efficacy endpoints (57), however, the *post-hoc* analysis of trial results revealed that patients treated with rivipansel shortly after VOC onset had benefited from the therapy. This led the FDA to grant a Rare Pediatric Disease designation for rivipansel to treat SCD in pediatric patients. Another glycomimetic, uproleselan (GMI-1271), a specific E-selectin inhibitor, is being investigated as an add-on therapy to standard chemotherapy in acute myeloid leukemia (58) and as antithrombotic treatment (59). Similarly, another anti-adhesive polysaccharide drug candidate, sevuparin (non-anticoagulant low molecular weight heparinoid), failed to improve clinical outcomes in patients with SCD (57, 60).

Interestingly, even though VCAM-1 is established as a viable target in immunological disorders and cancer (31), no human or humanized mAb or low molecular weight (Mw) inhibitor is being tested in clinics.

Research and development of these CAM inhibitors provided valuable insights on the complexity of future drug candidate development. Some of the lessons could be summarized as:

1. Inhibition of leukocyte infiltration by blocking CAMs is a viable strategy for limiting inflammation, as validated by the success of natalizumab and vedolizumab.
2. The clinical translation of several small molecule CAM inhibitors has stagnated either due to the insufficient binding affinity (glycomimetics), unfavorable pharmacokinetics (glycomimetics), lack of clinical efficacy (glycomimetics, anti ICAM-1, L-selectin, P-selectin mAbs) or potential immunogenicity (mAbs). Additionally, in the case of aselizumab it was postulated that even if the drug achieves the blockage of the majority of targeted CAMs, there is the possibility of compensatory activity of other CAMs that can diminish inhibitory effects on leukocyte infiltration.
3. Choosing the right pathological process (indication) matters. Blocking P-selectin was not successful in CTs for cardiovascular diseases, but later it proved to be a good strategy in a specific niche: VOC in SCD.

While the CAM-binding molecules alone showed insufficient efficacy, they could pose a functional part of a NM system. Using NM carriers with affinity ligands against CAMs could simultaneously provide both blocking of these leukocyte-anchors, which would potentially limit inflammation, and a possibility to deliver therapeutic cargo in a targeted manner right to the inflamed site. Attachment of multiple targeting ligands would improve the binding efficiency and the macromolecular nature of NM could improve the pharmacokinetic (PK) properties and stability, providing an answer to several issues that led to failure of mAbs and small molecule inhibitors of CAMs.

## Nanomedicines Targeting CAMs

In parallel with the development of mAbs and small molecule CAM inhibitors, many research groups aimed to develop CAM targeted NMs. Compared to small molecule CAM inhibitors and traditional small molecule anti-inflammatory or chemotherapeutic drugs, the advantages of NM are: a. better PK profile - prolonging half-life time in the circulation b. solubility enhancement of poorly water-soluble drugs c. improved stability d. delivery of the drug to the target site e. controlled release of the drug at the target tissue. Compared to mAbs, NMs that block CAM function possess: a. no/reduced immunogenicity b. multivalency c. the potential of steric shielding of cells. These goals can be achieved by the properties of NMs (macromolecular size and easy chemical modification) and can be further improved in order to target selected cells in the desired tissue by constructing NMs bearing multiple targeting ligands.

**Figure 1:**
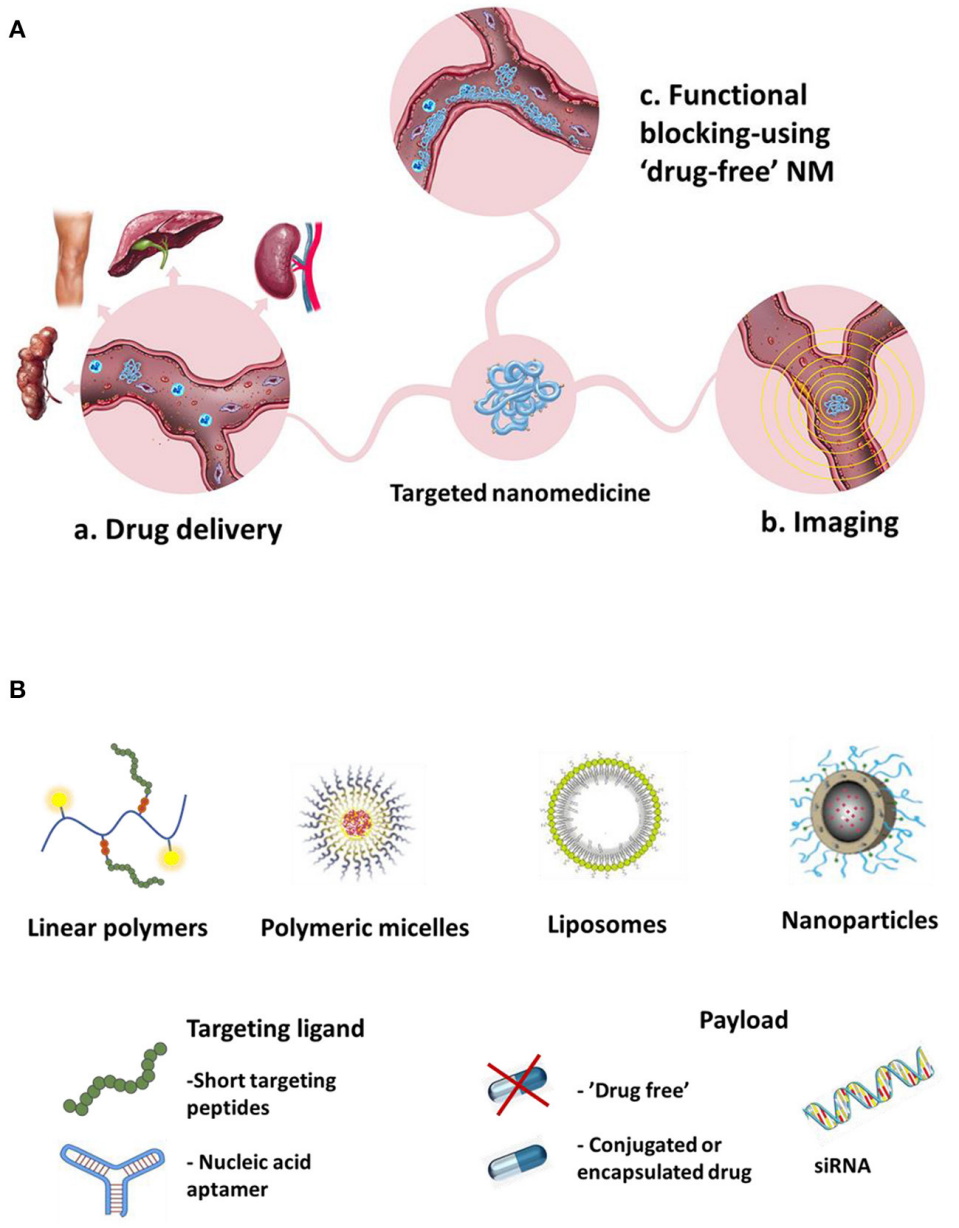
Different proposed goals of CAMs-targeted NM **(A)** Different systems used for targeting CAMs, types of targeting ligands and payloads **(B)**.

Targeting CAMs with affinity NMs can pursue several therapeutic goals (Figure 1A):

a. Targeted drug delivery to the inflamed endothelium - using CAM-targeted carriers bearing a. chemotherapeutic drugs to be delivered to the vicinity of tumors, or b. anti-inflammatory drugs for suppression of inflammation.
b. Imaging - using NM imaging probes targeted toward CAMs for imaging of a. cancer vasculature or b. inflamed vasculature.
c. Functional blocking - using “drug-free” NM (without cargo) to bind CAMs and block their function, in order to: a. inhibit leukocyte trafficking into the inflamed tissues or b. Inhibit cancer cells homing to the pre-metastatic niche and metastasis formation.

An array of different nanocarriers (e.g., liposomes, polymer-based NPs, magnetic NPs, dendrimers, quantum dots, Figure 1B) have been used for designing and constructing CAM-targeted NMs (61).

### a. Drug Delivery Systems

Localization of CAMs at the inflamed endothelium makes them attractive targets for drug delivery both to tumors and sites of inflammation of various pathologies. Accordingly, different CAM-targeted systems were developed to carry chemotherapeutic and anti-inflammatory drugs. Targeting ligands employed in these systems vary in their structural complexity, binding affinity, and potential to induce adverse effects. More details on the chemical/structural properties of these systems can be found in reviews by Muzykantov's group (61, 62).

One of the earliest approaches was to utilize oligosaccharides, the natural ligands of CAMs as ligands. Liposomes decorated with sLe^x^, a tetrasaccharide molecule present on the terminus of selectin glycoprotein ligands, were developed for selective binding to E-selectin and delivery of therapeutic agents to activated endothelium (63–65). Apart from targeting drugs to the tumor vasculature (66–68), such systems were used for the delivery of anti-inflammatory drugs in order to control inflammation in the eye or in inflamed joints (69, 70). In addition to liposomes, other drug-delivery systems (DDS) with conjugated sLe^x^ were reported, including polyethyleneoxide-b-polybutadiene (PEO-*b*-PBD) block copolymers and poly(d,l-lactide) (PLA) nanoparticles (71, 72). Due to the structural complexity of sLe^x^ and its complicated synthesis, several sLe^x^ mimicking structures (3'-(1-carboxy)ethyl (3'-CE)) or quinic acid (Qa)-based mimetics were used to decorate the surface of liposomes or were conjugated to an N-(2-hydroxypropyl)methacrylamide (HPMA) copolymer, respectively, as selectin targeting ligands, and exhibited higher uptake into activated endothelial cells (73, 74). The natural product polysaccharide fucoidan has also been proposed as a targeting ligand for P-selectin and employed in NM development, both for cancer drug delivery (75) and for inflammation control (76).

Monoclonal antibodies bind CAMs with higher affinity and selectivity compared to carbohydrate-based ligands, and therefore have been investigated as potential targeting moieties on DDS. Liposomes decorated with anti-E-selectin Ab were utilized for selective delivery of dexamethasone (DEX) to the inflamed kidneys in a murine model (77). Similar systems, targeting E-selectin or VCAM-1, were used for short interfering RNA (siRNA), cytotoxic (78, 79) or anti-inflammatory drug delivery (80). Anti-ICAM-1-targeted immunoliposomes were also utilized for delivery specifically to injured tissues in a rat paw inflammation model (81) and in lung injury model (82). To overcome the disadvantages of antibodies, such as the potential immunogenicity of the Fc fragment, and their relatively high molecular weight, some research groups have used antibody fragments as affinity ligands (61), however not many examples of advanced systems can be identified.

However, high “antibody-like” binding affinity can also be achieved with short targeting peptides. As NM ligands, peptides possess several advantages, mainly owing to their small size - low immunogenicity, stability, easy manufacturing and low cost (83). For example, our group utilized a short high-affinity E-selectin binding peptide (Esbp, DITWDQLWDLMK), identified in (84)) for targeted drug delivery of a cytotoxic drug (Doxorubicin, DOX) or a pro-apoptotic peptide (_D_(KLAKLAK)_2_, KLAK) to tumor vasculature. The hydrophilic HPMA-based copolymer bearing multiple copies of Esbp showed high binding affinity (at low nanomolar range) and selectivity to activated endothelial cells (85). The polymer-DOX conjugate (P-Esbp-DOX) demonstrated selective cytotoxicity toward E-selectin-expressing vascular endothelial cells that was 150-fold higher compared to a non-targeted polymer (85). *In vivo*, the E-selectin targeted polymer-drug conjugates (P-Esbp-DOX and P-Esbp-KLAK) decreased the rate of tumor growth and prolonged the survival of mice bearing primary Lewis lung carcinoma, or established melanoma (B16-F10) lung metastases (86). P-Esbp-DOX was also proven to be safe and highly efficacious in treating mice with established colorectal cancer liver metastases [unpublished data]. In addition to tumor targeting, this system was employed by our group for targeted delivery of an anti-inflammatory drug (DEX) to inflamed atherosclerotic plaques, to prevent cardiac remodeling and atherosclerosis (87). Esbp-modified bovine serum albumin (BSA) nanoparticles or Esbp-hyaluronic acid-paclitaxel (Esbp-HA-PTX) micelles were used by other groups to target DEX or PTX to acute lung injury or to inhibit breast cancer metastasis, respectively (88, 89). Other sequences such as IELLQAR (shown to bind to E-selectin and with much lower affinity to P- and L-selectin) (90–92), YRNWFGRW and YRNWDGRW (93) have been proposed as well for selective targeting of E-selectin. Interestingly, a study by Fernandes et al. directly compared all of these peptides for their ability to bind E-selectin. Their results suggest that Esbp is the ideal candidate for NM development, as it binds E-selectin better than the IELLQAR sequence and with better specificity when compared to the other two sequences (94).

Other short, high-affinity peptides were incorporated into NMs for targeting VCAM-1 [sequences VHPKQHR(GGSKGC) and cyclic FLDVRK (cyclo(MePhe-Leu-Asp-Val-D-Arg-D-Lys)) reviewed in (12)], P-selectin [sequences EWVDV (95–97) and LVSVLDLEPLDAAWL (98)] and ICAM-1 (cyclo(1,12)PenITDGEATDSGC (99) (Table 2).

**Table 2:** Selected examples of NM targeting CAMs for drug delivery.

**Class of targeting ligand**	**Targeting ligand**	**CAM**	**NM carrier**	**Payload**	**Site of delivery/purpose**	**Main result**	**References**
Carbohydrate-based	Sialyl Lewis^x^ (sLe^x^)	E-selectin, P-selectin	Liposomes, NPs	Various cytotoxic and anti-inflammatory drugs	Activated endothelium, cancer or inflamed tissues	Preferential drug delivery to activated endothelium	(63–70)
	3'-(1-carboxy)ethyl (3'-CE)	E-selectin	Liposomes	/	Inflamed endothelium (HUVEC)	Higher internalization by huvecs	(73)
	Quinic acid (Qa) based sLe^x^ mimetic	E- and P-selectin	HPMA copolymer	/	Inflamed endothelium	Selective binding and higher internalization by IVECS	(74)
	Fucoidan	P-selectin	dextran sulfate-based NPs	BYL719 (PI3Kα inhibitor)	Tumor microenvironment	Tumor growth suppression, Reduction of systemic adverse effects of BYL719	(75)
mAbs	Anti-E-selectin Ab	E-selectin	Liposomes	DEX	Inflamed kidney	Reduced inflammation *in vivo*	(77)
	anti-VCAM-1	VCAM-1	Liposomes based formulation-LipoCardium	PGA_2_	Atherosclerotic plaque in LDL receptor knock-out mice	Reduction in plaque progression, reduced death due to MI	(80)
	Anti-ICAM-1 Ab	ICAM-1	Liposomes	LOP	Inflamed rat paw	Better pain control, *in vivo*	(81)
				DEX	Lung endothelium	Reduced lung inflammation	(82)
Peptides (primary sequence)^*^	DITWDQLWDLMK	E-selectin	HPMA-based polymer	DOX, _D_(KLAKLAK)_2_ or DEX	Tumor vasculature, atherosclerotic plaque in ApoE-/- mice	Tumor growth reduction, inhibition of metastases, plaque stabilization	(85–87)
			BSA-based NPs	DEX	Acute lung injury	Better accumulation in the inflamed region	(88)
			HA-PTX micelles	PTX	Breast cancer lung metastasis	Inhibition of tumor growth and metastasis, and decreased systemic toxicity	(89)
	IELLQAR	E-selectin	Self-assembled NPs	SN38-metabolite of irinotecan	Tumor endothelium	Inhibition of tumor growth, “drug-free” NP reduced the metastases formation	(92)
	VHPKQHR	VCAM-1	Liposomes, lipoparticles, micelles, lipid nanoemulsions	CCR2 Antagonist, flavonoids	Inflamed (tumor) endothelium, atherosclerotic plaque in ApoE-/- mice	Inhibition of metastases in mice models, reduced transmigration of monocytes, atherosclerosis inhibition	(12, 100–102)
	Cyclic FLDVRK (mZD7349 peptide)	VCAM-1	PLGA NPs	SIM	Activated HUVEC	Improved uptake into HUVEC, decreased phosphorylation of eNOS	(103)
	EWVDV	P-selectin	Magnetic Fe_3_O_4_ NPs	/	Binding to platelets that can accumulate in breast cancer tumors	Platelets targeting was successful in breast cancer, but not in pancreatic cancer models	(97)
			Micelles	PTX	Targeting circulating platelets, for delivery to CTC and primary tumor	Suppression of lung metastases in TNBC model	(95)
			Lipid NPs	Ticagrelor and celecoxib	Targeting platelets	Accumulation in tumor via capturing platelets, antimetastatic effects	(96)
	LVSVLDLEPLDAAWL	P-selectin	Lipid nanoemulsions	DEX	Inflamed lungs	Accumulation of NM in the inflamed lungs, reduction of inflammation (cytokines expression)	(98)
	Cyclo(1,12) PenITDGEATDSGC (cLABL)	ICAM-1	PLGA NPs	DOX	Binding to activated HUVEC, Lung epithelial cells	Delivery of DOX to ICAM-1 expressing cells	(99)
However, high “antibody-like” binding affinity can also be achieved with short targeting peptides. As NM ligands, peptides possess several advantages, mainly owing to their small size - low immunogenicity, stability, easy manufacturing and low cost (83). For example, our group utilized a short high-affinity E-selectin binding peptide (Esbp, DITWDQLWDLMK), identified in (84)) for targeted drug delivery of a cytotoxic drug (Doxorubicin, DOX) or a pro-apoptotic peptide (_D_(KLAKLAK)_2_, KLAK) to tumor vasculature. The hydrophilic HPMA-based copolymer bearing multiple copies of Esbp showed high binding affinity (at low nanomolar range) and selectivity to activated endothelial cells (85). The polymer-DOX conjugate (P-Esbp-DOX) demonstrated selective cytotoxicity toward E-selectin-expressing vascular endothelial cells that was 150-fold higher compared to a non-targeted polymer (85). *In vivo*, the E-selectin targeted polymer-drug conjugates (P-Esbp-DOX and P-Esbp-KLAK) decreased the rate of tumor growth and prolonged the survival of mice bearing primary Lewis lung carcinoma, or established melanoma (B16-F10) lung metastases (86). P-Esbp-DOX was also proven to be safe and highly efficacious in treating mice with established colorectal cancer liver metastases [unpublished data]. In addition to tumor targeting, this system was employed by our group for targeted delivery of an anti-inflammatory drug (DEX) to inflamed atherosclerotic plaques, to prevent cardiac remodeling and atherosclerosis (87). Esbp-modified bovine serum albumin (BSA) nanoparticles or Esbp-hyaluronic acid-paclitaxel (Esbp-HA-PTX) micelles were used by other groups to target DEX or PTX to acute lung injury or to inhibit breast cancer metastasis, respectively (88, 89). Other sequences such as IELLQAR (shown to bind to E-selectin and with much lower affinity to P- and L-selectin) (90–92), YRNWFGRW and YRNWDGRW (93) have been proposed as well for selective targeting of E-selectin. Interestingly, a study by Fernandes et al. directly compared all of these peptides for their ability to bind E-selectin. Their results suggest that Esbp is the ideal candidate for NM development, as it binds E-selectin better than the IELLQAR sequence and with better specificity when compared to the other two sequences (94). Other short, high-affinity peptides were incorporated into NMs for targeting VCAM-1 [sequences VHPKQHR(GGSKGC) and cyclic FLDVRK (cyclo(MePhe-Leu-Asp-Val-D-Arg-D-Lys)) reviewed in (12)], P-selectin [sequences EWVDV (95–97) and LVSVLDLEPLDAAWL (98)] and ICAM-1 (cyclo(1,12)PenITDGEATDSGC (99) (Table 2). Similar to short peptides, short sequences of nucleotides, commonly referred to as DNA or RNA aptamers were also developed for targeting CAMs. A thioaptamer targeting E-selectin (ESTA-1) (109) demonstrated selective binding with nanomolar binding affinity, and was able to target porous silicon particles to the tumor vasculature in a breast cancer xenograft model, endothelium of bone marrow or atherosclerotic plaque (104–106). Conversely, other groups reported that they couldn't confirm ESTA-1 binding to human E-selectin in their assays (110) and identified a new high affinity E- and P-selectin binding aptamer (SDA). These discrepancies could serve as a warning of difficulties in the translation of these ligands among different systems, laboratory facilities, and types of NM. CAM-targeting of NM can also be achieved through usage of cell-membrane fragments of cells known to engage with CAMs. Liang et al. (107) used the presence of α4 integrin on the surface of macrophages to generate membrane-coated liposomes that could bind to VCAM-1 and effectively deliver drug payload to lung metastases. Recently, a new approach for generating targeting ligands on NMs emerged from the advances in genetical engineering. Park et al. (108) developed VCAM-1 targeted NM by coating poly(lactic-co-glycolic acid) (PLGA)-based NPs with membrane fragments of cells expressing the ligand for VCAM-1. They chose a cell line which expresses β_1_ integrin, and modified it to express integrin α_4_, which together form a complex, VLA-4, a ligand for VCAM-1. This NM was loaded with DEX and used for drug delivery to inflamed lungs and suppressing inflammation. Aptamers	ESTA-1	E-selectin	Porous silicon microparticles	PTX, microRNA	Bone marrow, xenograft breast tumors, atherosclerotic plaque	Bone marrow accumulation, breast cancer targeting. Reduced endothelial inflammation	(104–106)
Cell-membrane fragments	Macrophage-derived cell membranes	VCAM-1	Liposomes	DOX	Lung metastases	Improved lung metastases imaging and tumor growth inhibition	(107)
	Genetically engineered cell-membrane fragments	VCAM-1	PLGA NPs	DEX	Inflamed lungs	Reduction of inflammation (cytokine levels)	(108)

*DOX-doxorubicin (hydrochloride) DEX-dexamethasone PTX-paclitaxel LOP-loperamide SIM-simvastatin HUVEC-human umbilical vein endothelial cells MI-myocardium infarction*.

*eNOS- endothelial NO synthase*.

* *Peptide sequences are presented in the primary binding form, without spacers and amino acids used for conjugation to NM*.

Similar to short peptides, short sequences of nucleotides, commonly referred to as DNA or RNA aptamers were also developed for targeting CAMs. A thioaptamer targeting E-selectin (ESTA-1) (109) demonstrated selective binding with nanomolar binding affinity, and was able to target porous silicon particles to the tumor vasculature in a breast cancer xenograft model, endothelium of bone marrow or atherosclerotic plaque (104–106). Conversely, other groups reported that they couldn't confirm ESTA-1 binding to human E-selectin in their assays (110) and identified a new high affinity E- and P-selectin binding aptamer (SDA). These discrepancies could serve as a warning of difficulties in the translation of these ligands among different systems, laboratory facilities, and types of NM.

CAM-targeting of NM can also be achieved through usage of cell-membrane fragments of cells known to engage with CAMs. Liang et al. (107) used the presence of α4 integrin on the surface of macrophages to generate membrane-coated liposomes that could bind to VCAM-1 and effectively deliver drug payload to lung metastases.

Recently, a new approach for generating targeting ligands on NMs emerged from the advances in genetical engineering. Park et al. (108) developed VCAM-1 targeted NM by coating poly(lactic-co-glycolic acid) (PLGA)-based NPs with membrane fragments of cells expressing the ligand for VCAM-1. They chose a cell line which expresses β_1_ integrin, and modified it to express integrin α_4_, which together form a complex, VLA-4, a ligand for VCAM-1. This NM was loaded with DEX and used for drug delivery to inflamed lungs and suppressing inflammation.

### b. Imaging

Targeting of imaging probes to endothelial CAMs in cancerous and inflammation sites holds promise to improve management of these conditions. Nano-sized imaging probes of diverse shape, materials and physical properties have been developed over the years to improve the visualization of pathological sites by intravital microscopy (IVM), positron emission tomography (PET), magnetic resonance imaging (MRI) and ultrasound (US) modality in pre-clinical settings (111). Selected examples of CAMs-targeted NM imaging probes are listed in Table 3. In principle, imaging of vascular inflammation can be easily achieved with ligand-targeted imaging probes, since CAMs on the activated endothelium are readily accessible for circulating nanomaterials.

**Table 3:** Selected examples of NM targeting CAMs for Imaging.

**Class of targeting ligand**	**Targeting ligand**	**CAM**	**Material/NM carrier**	**Imaging modality**	**Main result**	**References**
Ab	Anti-VCAM-1 Ab	VCAM-1	35 nm CLIO magneto-optical particles	IVM	Accumulation at the site of inflammation in a mouse ear	(112)
	Anti-αvcam-1 (clone A(429)	VCAM-1	MPIO- DynaBeads	MR	Imaging of early atherosclerosis and microcalcifications	(113)
	Anti-E-selectin Ab	E-selectin	USPIO (30–50 nm)	MR	Contrast enhancement in TNF-induced inflammation of the mouse ear	(114)
	Anti-ICAM-1	ICAM-1	^64^Cu-labeled latex nanoparticles (NPs)	PET	Accumulation in anti-ICAM/NPs in the inflamed lungs of LPS-challenged mice	(115)
Ab fragments	Anti-human E-selectin (CD62E) F(ab‘)2 fragments	E-selectin	CLIO	MR	Binding to activated HUVECs	(116)
Carbohydrate based ligands	sLe^X^	E- and P-selectin	35 nm CLIO	MR	Detection of E- and P-selectin in a multiple sclerosis rat model -pre-symptomatic brain imaging	(117)
	Mimetic of sLe^x^	E-selectin	Dextran coated USPIO	MR	Binding to HUVECs and to activated liver endothelium	(118)
	Qa	P-selectin	USIONPs <4 nm	MR	Binding and uptake into cancer cells	(119)
Targeting peptides (primary sequences)	VHSPNKK (VHS)	VCAM-1	Magneto-fluorescent NP, termed VNP	Intravital confocal microscopy, MR	Identification of peptide sequence by phage display. 12-fold higher binding relative to anti-VCAM-1 Ab *in vitro*. Binding to atherosclerotic lesions in ApoE-/- mice	(120, 121)
			Simian virus 40 (SV40)-based nanoparticles with QD	Fluorescence imaging	Imaging of atherosclerosis in live ApoE(-/-) mice	(122)
	VHPKQHR (VINP-VCAM-1 internalizing peptide-28)	VCAM-1	Magneto-fluorescent NP, CLIO-Cy5.5 termed VINP28	MR and optical imaging	20 times higher affinity for VCAM-1 than previously reported for VNP. Imaging of atherosclerotic mice and human atheroma	(121, 123)
			MB	Ultrasound	MC38 murine colon Adenocarcinoma imaging	(124)
	RANLRILARY (B2702-p)	VCAM-1	Microbubbles	Ultrasound	VCAM-1 is significantly upregulated in symptomatic patients with ath. plaques. MB binding to aortic endothelial cells.	(125)
	DITWDQLWDLMK (Esbp)	E-selectin	Microbubbles	Ultrasound	Imaging of ischemic myocardium	(126)
			HPMA-based copolymer	Fluorescence imaging	Detection of atherosclerotic lesions in ApoE-/- mice	(87)
	IELLQAR	E-selectin (P- and L-selectin)	Microbubbles	Ultrasound	Binding to HUVECs under flow conditions accumulation in tumor vasculature *in vivo*	(127)
	LVSVLDLEPLDAAWL	P-selectin	50 nm dextran iron oxide particle	MR	3-fold higher accumulation in in infarcted tissue in ischemia/reperfusion brain injury	(128)
	EWVDV	P-selectin	Poly (lactic-co-glycolic acid) (PLGA)	Ultrasound, MR	Targeting and imaging of thrombi in models of different blood vessels under blood flow. *In vivo*: thrombus rabbit model	(129, 130)
Aptamers	Anti-VCAM-1 ssDNA full length (11R6) or truncated (A11R6)	VCAM-1	SPIO	Noninvasive BLI, MR	This theranostic NM was shown to bind to tumor cells in inhibit tumor growth *in vivo*, in addition to providing diagnostic tool	(131)
	E-selectin thioaptamer (ESTA-1)	E-selectin	A variety of different-sized silicon particles	MR	Imaging upon direct injection into ovarian cancer in a orthotopic mouse model	(132)

*BLI, bioluminescence imaging; CLIO, crosslinked iron oxide; MPIO, microparticles of iron oxide; MB, microbubbles; MR, magnetic resonance; PET, positron emission tomography; SPIONs, Superparamagnetic nanoparticles of iron oxides; USPIO, ultrasmall superparamagnetic iron oxide particles; USIONPs, Ultrasmall iron oxide nanoparticles*.

Indeed, a variety of NM systems were developed for diagnostic purposes. These molecular probes are designed to bind to CAMs allowing early detection of inflammatory lesions, and metastases formation, as well as disease progression and treatment monitoring. Most of such systems are directed at imaging of atherosclerotic lesions. VCAM-1 is the most exploited target, followed by E- and P-selectin (Table 3). As seen with NMs for drug delivery, first systems used mAbs and carbohydrates as targeting ligands. In more recently developed systems, researchers shifted to using short high-affinity targeting peptides or in a few cases, nucleotide aptamers, due to the already discussed favorable characteristics. The best example for this development could be the evolution of VCAM-1 targeting. Motivated by the sub-optimal performances of mAb targeted NMs for VCAM-1 imaging, Kelly et al. (120) employed a modified phage display approach, identifying a panel of binding peptides. One of the sequences with the highest binding affinity, a cyclic peptide VHSPNKK (VNP), was homologous to very late antigen-4 (VLA_4_), a counterpart of VCAM-1. *In vitro*, the peptide was shown to bind to VCAM-1 with 12-fold higher target-to-background ratios comparing to anti-VCAM-1 mAb. Magneto-fluorescent NPs modified with this peptide were tested *in vivo*. Interestingly, in their following work, the authors conducted further phage-display studies with *in vivo* atherosclerotic plaques, and identified a linear sequence VHPKQHR (VINP), with even higher binding affinity. In direct comparison of VNP and VINP, the latter was shown to bind VCAM-1 with 20 times higher affinity (123). After conjugation to magneto-fluorescent NP, this NM demonstrated excellent binding and uptake into endothelial cells and signal enhancement in a TNFα inflammation model, atherosclerotic ApoE^−/−^ mice and human atheroma samples.

### c. Functional Blockade of Immune and Cancer Cell Homing

The proinflammatory and disease promoting function of CAMs, their upregulation and hence increased impact on the course of disease have placed CAMs among viable targets for controlling pathological cell migration processes by functional receptor blockade. Accomplishments with mAbs and small molecule inhibitors are briefly summarized in this review.

However, the CAM-blocking properties of NMs targeted to CAMs have just recently entered the spotlight of scientific interest. The presence of targeting ligands on NMs can be utilized not only to direct a drug or dye cargo, but to functionally block CAM-mediated activity. These “drug-free macromolecular therapeutics” present an alternative to the discussed small molecule inhibitors and Abs recognizing CAMs and are summarized in Table 4.

**Table 4:** Selected examples of “drug-free” NM targeting CAMs.

**Class of targeting ligand**	**Targeting ligand**	**Targeted CAM**	**NM carrier**	**Implication/disease model**	**Reference**
Carbohydrate	sLe^X^/sLe^a^	E-selectin	Polyacrylamide (PAA)	Cell-free binding assays demonstrate a ~200 fold lower IC_50_ of NM compared to monovalent ligands	(133)
	sLe^X^/sLe^a^ (+tyrosine sulfate)	E-selectin (P-/L-selectin)	N-(hydroxyethyl)acrylamide	Ligand concentration dependent binding to E-selectin (and binding to P- and L-selectin) Inhibition of HL60 cell adhesion to E-selectin was increased	(134)
	sLe^X^/ galactose, fucose, sialic acid tyramine sulfate	E-, P- and L-selectin	HPMA	Multivalent presentation of sLe^X^-mimicking carbohydrates and sulfate groups effectively binds macrophages and reduces migration	(135)
	Mannose	P-selectin	PAA	Inhibition of neutrophil infiltration in rat peritoneal inflammation	(136)
	Sulfate groups	L-selectin	Dendritic polyglycerol	L-selectin and leukocyte rolling inhibition increase with size and degree of sulfation of dendritic polyglycerol sulfate	(137)
	Sulfate groups	P-/L-selectin	Dendritic polyglycerol	Reduced neutrophil infiltration in acute allergic contact dermatitis mouse model	(138)
	Sulfated β-lactose	L-selectin	Dendritic polyethylene oxide	Inhibition of neutrophil and macrophage infiltration in peritoneal inflammation in mice	(139)
	sLe^X^/ galactose, fucose, sialic acid tyramine sulfate	E-, P- and L-selectin	HPMA	Multivalent presentation of selectin-ligands effectively binds leukocytes and inhibits toxic liver injury	(140)
	Sulfate groups	P-/L-selectin	Dendritic polyglycerol	Decreased severity of disease in a murine model of polymyositis, reduced infiltration of pro-inflammatory T-cells	(141)
Peptide	E-selectin binding peptide DITWDQLWDLMK	E-selectin	HPMA	Inhibition of neutrophil adhesion under shear stress, reduction of alcohol-induced liver injury	(142)
	E-selectin binding peptide DITWDQLWDLMK	E-selectin	HPMA	Inhibition of high-fat diet promoted cardiac remodeling and reduction of atherosclerotic lesions in ApoE^−/−^ mice	(87)
	IDLMQARGC IELLQARGC QITWAQLWNMMKGC	E-/P-selectin (L-selectin, ICAM-1)	Dermatan sulfate	Reduction of neutrophil arrest to endothelial cells and migration, reduced thrombus formation in deep vein thrombosis mouse model	(90, 143)
	Esbp DITWDQLWDLMK	E-selectin	HPMA	Prevention of B16-F10 melanoma lung metastasis	(86)
	CD44 binding peptide KRLVSYNGIIFFLR	CD44v3/CD44v6	HPMA	Prevention of 4T1 breast cancer lung metastasis	(144)
	ITDGEATDSG	ICAM-1	Poly-DL-lactic-co-glycolic acid	Blockade of DC and T-cell interaction, arrest of T-cell proliferation	(145)
Aptamer	Anti-VCAM-1 aptamer	VCAM-1	SPIONs	*In vivo* anti-tumor activity when combined with anti-IL-4Rα aptamer conjugated SPIONs	(131)

The choice of carrier systems and ligands, as well as the design of ligand presentation, determine the blocking efficacy. Especially the multivalent presentation of ligands such as carbohydrates, which often possess relatively weak binding affinity, displays an advantage over small molecules or mAbs, which have only one or few binding sites (146, 147). The significantly higher binding affinity achieved by multiple copies of the selectin ligands sLe^X^ or sLe^a^ conjugated to a linear backbone, such as polyacrylamide (PAA) or HPMA, compared to monovalent sLe^X^/sLe^a^, illustrates the potential of multivalent systems to target CAM-expressing cells more efficiently than their monovalent counterparts (133–135). The same concept applies to other, non-natural ligands such as glycomimetic (134–136) or non-carbohydrate ligand analogs (Table 4) (74, 85).

Besides higher binding affinity, the use of NMs for functional blockade reveals another advantage over non-polymeric inhibitors, which is the shielding from cell-cell interactions by nanosized inhibitors. The binding of CAMs to ligands on flexible polymeric carriers sterically hinders the interaction of surrounding adhesion molecules with their ligands and therefore increases the inhibitory potential of the NM (137, 148, 149). This was extensively investigated for P- and L-selectin targeted dendritic polyglycerol sulfates (dPGS), for which increasing size, as well as increasing valency, are associated with higher inhibitory activity (137, 138). Similar dPGS system was also employed for drug delivery, through binding to P-selectin on both the activated endothelium and cancer cells expressing high levels of this marker (glioblastoma multiforme) (150).

Indeed, several of these “drug-free” CAM-targeted NMs have proven their therapeutic potential in pre-clinical research (147, 151, 152). sLe^X^- or other glycomimetic ligand-bearing polymer treatment reduces neutrophil and/or macrophage infiltration in rodent models of inflammation, such as peritoneal inflammation (136, 139), liver injury (140) and dermatitis (138). In a murine model of polymyositis, the infiltration of pro-inflammatory T-cells was blocked by dPGS, targeting their L-selectin mediated recruitment (141).

High-affinity peptides as targeting moieties, have been investigated for targeted drug delivery and imaging, but also possess desirable properties for the functional blockade of CAMs. In our research, we demonstrated that an HPMA-based copolymer carrying multiple copies of Esbp inhibits neutrophil capture and endothelial transmigration (142). The strong inhibition of E-selectin function, without the involvement of anti-inflammatory drugs, is an effective strategy to reduce alcohol-induced liver injury (142) or high fat diet-promoted cardiac remodeling (87) in apolipoprotein E knockout (ApoE^−/−^) mice. P-Esbp reduced atherosclerotic lesions in these mice, stabilized atherosclerotic plaques and reduced the total leukocyte count in the blood stream, as a response to less inflammation (87). Similarly, P-Esbp treatment caused a reduction of E-selectin expression in inflamed livers in mice (post ethanol feeding), indicating a sustainable reduction of inflammation (142). Along in the same lines stand results of neutrophil adhesion blockade achieved by three different peptides, respectively attached to a dermatan sulfate backbone, binding to E- and P-selectin, as well as, to a lesser extent, to L-selectin and ICAM-1 (90, 143). These glycopolymer-peptide conjugates reduce thrombus size in a deep vein thrombosis model by inhibiting platelet activation (143).

In our other studies, we have shown that P-Esbp effectively inhibits the cell-cell interaction with E-selectin ligand-expressing B16 melanoma cells (86). A pretreatment with P-Esbp prevented the extravasation and tissue colonization of circulating cancer cells, which resulted in a significant decrease of established lung metastasis colonies in mice (86). Accordingly, we have designed an HPMA copolymer bearing the laminin α5-derived peptide A5G27 (P-A5G27), which specifically binds CD44v3 and CD44v6, two subtypes of the selectin ligand CD44, which are expressed on invasive cancer cells (144). We confirmed that P-A5G27 reduces cell migration *in vitro* and inhibits cancer cell metastasis of murine 4T1 breast cancer cells *in vivo*.

Blocking of ICAM-1 by the short cyclic peptide cLABL, was investigated for interfering with immunological synapses between T-cells and antigen presenting dendritic cells (DCs). Binding of PLGA NPs decorated with cLABL decreased T-cells conjugation to DCs, inhibiting the activation of T-cells, much more effectively than the free cLABL peptide (145).

Already discussed anti-VCAM-1 aptamer conjugated SPIONs, apart from demonstrating favorable tumor imaging ability, were also investigated for tumor growth suppression (131). When injected along with anti-IL-4Rα aptamer-conjugated SPIONs to tumor bearing mice, these two NMs significantly decreased tumor size comparing to control or single NM-treated animals, indicating that simultaneous functional blockade of these targets potentiates antitumor activity. Similarly, ESTA-1 thioaptamer was shown to block adhesion of sLe^X^ positive cells to endothelium (109). However, in addition to drug delivery this thioaptamer is being developed as a stand-alone treatment (153), probably owing to the improved serum stability and to our best knowledge no ESTA-1 drug-free NM was reported.

### d. Other Vascular-Targeted Nanomedicines

A separate class of CAMs are integrins, a group of heterodimeric transmembrane receptors (composed of one α- and one β-subunit) with various functions in cell-cell and cell-extracellular matrix interactions. This family of receptors can be divided into several groups, based on their ligand specificity: endothelial specific receptors, leukocyte-specific receptors, laminin-binding receptors, and collagen-binding receptors (154). In contrast to the other groups of discussed CAMs, integrin expression is not limited to the endothelium, but they are present inside many other tissues, and their expression on the leukocyte surface makes them another regulator of leukocyte infiltration (155).

RGD-binding integrins were named for their affinity to tripeptide Arginine-Glycine-Aspartic acid (RGD) and are present at elevated levels on a variety of different cancer cells (156). Their blockade via RGD-based ligands has been demonstrated beneficial for cancer therapy, and a great variety of NMs with RGD-based targeting are extensively reviewed elsewhere (155, 157, 158). In order to improve binding affinity, selectivity and stability, several modifications of RGD peptides were reported, including cyclization, incorporation of D-amino acids and N-methylation (156). While many integrins are involved in the regulation of vascular functions, only few are predominantly expressed on the vascular endothelium (159, 160) therefore only a small number of systems is designed to target specifically endothelium expressed α_v_β_3_ and α_v_β_5_ integrins (Table 5). Integrin α_v_β_3_ and α_v_β_5_ are upregulated on the tumor neovasculature and critically associated with angiogenesis. In addition to discontinued mAb drug candidate, Etaracizumab (166), several targeted nanomedicine were developed for imaging and drug delivery purposes. Earliest attempts include using the anti-α_v_β_3_ antibody LM609 conjugated to the surface of paramagnetic liposomal nanoparticles for tumor imaging (162) or utilizing small molecule integrin α_v_β_3_ inhibitor on the surface of cationic nanoparticles for gene delivery to tumor endothelium (163). Through lead optimization of the integrin antagonist compounds, Xie et al. developed an antagonist with superior binding to the previously used one and conjugated it in a multiple manner to lipid nanoparticles. These NPs accumulated in the tumor angiogenic vessels in a murine model of squamous cell carcinoma (164).

**Table 5:** Selected examples of NMs targeting endothelial integrins.

**Targeting ligand**	**Carrier**	**Aim**	**Target**	**References**
RGD	Various	Theranostic, inhibition of angiogenesis by blocking integrin function	α_v_β_3_/ α_v_β_5_	Reviewed (155, 157, 158)
iRGD	Various	Imaging and image-guided phototherapy	α_v_β_3_/ α_v_β_5_	Reviewed (161)
Antibody LM609	Paramagnetic liposomal nanoparticles	Imaging	α_v_β_3_	(162)
Small molecule integrin α_v_β_3_ inhibitor	Cationic lipid nanoparticles	Gene delivery	α_v_β_3_	(163)
Integrin α_v_β_3_ binding compound (IA)	Lipid nanoparticles	Theranostic	α_v_β_3_	(164)
HSDVHK	Micelles	Inhibition of angiogenesis	α_v_β_5_	(165)

Several drug-loaded nanoparticles decorated with cyclic iRGD (CRGDKGPD) peptides have been investigated as DDS for cancer therapy, which bind to vascular integrins and additionally to neuropilin-1 (161). iRGD-decorated indocyanine green-loaded liposomes, which target endothelial integrins α_v_β_3_ and α_v_β_5_, were developed for imaging and imaging-guided phototherapy (167).

Besides cancer therapy, DDS targeting α_v_β_5_ via RGD-peptide have demonstrated therapeutic antiangiogenic efficacy for the treatment of choroidal neovascularization (168). Recent *in vitro* results confirm the contribution of a multivalent ligand presentation for the inhibition of angiogenesis, using drug-free antiangiogenic peptide GHSDVHK-decorated micelles (165).

## Strategies and Considerations for Designing Cam-Targeted NMs

Whether or not an actively targeted NM will demonstrate high selectivity for the diseased tissue, will depend on the binding avidity of the NM toward its target and on the selective localization and the degree of up-regulation of the target itself. Certain CAMs can be present at un-desired locations (selectins in skin or bone marrow or on platelets) or are insufficiently up-regulated at the desired site (8, 11). Increasing the site-specific accumulation of NMs can sometimes be achieved by strategical use of dual or simultaneous targeting or enhancement of CAM expression.

### a. Dual or Simultaneous Targeting of Multiple CAMs

If we consider the physiological functions of CAMs in leukocyte adhesion and tissue transmigration, it is intuitively apparent that binding multiple CAMs can ensure better affinity of NMs, leading to superior efficacy. The notion of potential binding synergism mainly was investigated in molecular ultrasound imaging by targeted microbubbles (MBs) (169, 170).

Weller et al. reported the development of ultrasound contrast MBs with targeting moieties against both ICAM-1 (anti-human-ICAM-1 antibody) and selectins (sLe^X^) with the aim to improve imaging of inflamed vasculature. These dual-targeted MBs demonstrated 20 % higher adhesion to activated endothelial cells under flow, than MBs bearing either anti-ICAM-1 Ab or sLe^X^ alone (169). Similarly, dual-targeted MBs bearing mAb against VCAM-1 and a sLe^X−^decorated PAA polymer for selectin targeting were developed by Ferrante et al. In a series of flow chamber experiments, the dual-targeted MBs were compared to single targeted microbubbles. At higher shear stress conditions, the dual-targeted MBs out-performed both of the single-targeted MBs (170). Additional steps toward obtaining ‘leukocyte-like' NMs were achieved by Yan et al. who developed VCAM-1/ICAM-1/selectin targeted MBs by integrating VCAM-1 and ICAM-1 antibodies, and synthetic polymeric sLe^x^ onto the MBs surface. Triple-targeted MBs achieved 3-fold higher binding to activated endothelial cells compared to single and dual-targeted MBs. *In vivo* data confirmed the advantageous effects of triple-targeting in ultrasound molecular imaging of atherosclerotic plaques of ApoE-deficient mice (171).

In addition to imaging, multiple-targeting was proposed for general drug delivery to the inflamed endothelium. Using model polystyrene particles decorated with sLe^X^ and/or anti-ICAM-1 Ab, Fromen et al. confirmed the synergy of dual targeting and demonstrated 3-7-fold increase in adherence compared to single-targeted particles (172).

Another example of multiple targeting leukocyte-mimicking particles are polymerosomes - synthetic analogs of leukocytes assembled from block copolymers and functionalized for simultaneous targeting of ICAM-1 and selectins, by anti-ICAM-1 and sLe^x^, respectively (71). More recently, lipid NPs incorporating leukocyte plasma membrane proteins termed leukosomes were developed. These structures were shown to preferentially target inflamed murine endothelium in models of LPS-induced ear inflammation (173, 174), atherosclerotic plaques or breast cancer (175) and were able to deliver DEX to the tissues (173). Among other cell membrane proteins, ligands for ICAM-1, VCAM-1, E- and P-selectin were incorporated in the leukosome lipid bilayer and were crucial for tumor accumulation (175).

### b. Enhancement of CAM Expression With Pharmacological or Physical Agents

#### Radiation

Radiation therapy has been proven to be an effective modality in cancer treatment, however, it is non-specific, and its effects cannot be limited to malignant cells. Radiation-induced damage to the surrounding endothelium can in fact change the elasticity and functionality of cancer vasculature (176), increase the vascular permeability (177) and activate endothelial cells causing an overexpression of various CAMs (178–181). Apart from *in vitro* and mouse model data, these changes were confirmed in cancer patients (182).

This change in the expression of endothelial CAMs was exploited to further enhance the targeting of cancer vasculature by NMs. First systems including anti-ICAM-1 targeted microspheres (183) or iron oxide microparticles (184) showed several folds higher binding to irradiated endothelial cells compared to control cells. Anti-E-selectin antibody-conjugated immunoliposomes were utilized for the delivery of the vascular-damaging drug combretastatin A4, to MCa-4 mouse mammary tumors (185). The basal expression of E-selectin in these tumors was shown to be low, however, it was significantly upregulated after irradiation, allowing for the preferential delivery of immunoliposomes and tumor growth delay.

Radiation-guided endothelial targeting can be achieved with other targeting ligands, besides antibodies. Quinic acid analogs as synthetic mimetic of selectin ligands, were used for decorating PLGA nanoparticles (Qa-NP), yielding NMs which bind to E- and P-selectin (186). To investigate biodistribution, mice bearing CT26 colon cancers in both hind limbs underwent treatment of 6 Gy X-irradiation in only one tumor. Confocal imaging with immunofluorescence staining confirmed the upregulation of E- and P-selectin in irradiated tumors and greater accumulation of QA-NP. Polysaccharide fucoidan, which exhibits nanomolar affinity to P-selectin, was utilized for preparing a nanoparticle carrier for a chemotherapeutic drug. When injected in mice bearing bilateral flank LLC tumors, this system exhibited 3.8-fold higher fluorescence signal in irradiated over non-irradiated tumors (187). Interestingly, an increase in P-selectin expression was also detected in non-irradiated tumors, however to a lesser extent than in irradiated ones, indicating an immune system-mediated activation of distant sites.

Apart from radiation, other physical agents, such as heat were shown to induce up-regulation of CAMs mostly in the tumor vasculature (188), without affecting non-activated endothelium (189). However, we did not identify specific NMs which exploit this approach.

#### Pharmacological Agents

Considering the inflammation-inducible nature of CAMs, the most obvious intervention that could enhance their presence on the blood vessel endothelium is administration of pro-inflammatory cytokines. However, systemic administration of such cytokines would lead to overall inflammation and, given their complex effects on many receptors and multiple tissues, unpredictable adverse effects. For example, TNFα, although named for its ability to shrink certain tumors in animal models, turned out to have a rather complex effect on tumor growth and a potential for supporting tumor progression (190). Furthermore, systemic administration of TNFα induced a dose limiting toxicity, a condition termed “septic shock like syndrome” (21, 191).

A more tailored approach includes using known drugs, with defined safety profiles that have been shown to up-regulate CAMs at the sites of inflammation. Imiquimod, an agonist of toll-like receptor 7 (TLR-7) was shown to induce E-selectin expression on previously negative human squamous cell carcinomas (SCC) (21, 192), which promoted T-cell infiltration. This finding could be employed for directing E-selectin targeted NMs to tumor vasculature, but given that imiquimod is a topical drug further modalities of application should be investigated in order to cover a broader spectrum of applications.

Another pharmacological intervention exploits the connection between CAM expression and angiogenesis. As it is known that angiogenic factors inhibit the expression of CAMs (ICAM-1 and VCAM-1) (21), some studies looked into effects of angiogenesis inhibitors on vascular CAM expression. Anginex, a synthetic anti-angiogenic peptide-drug candidate, significantly reduced the downregulation of ICAM-1, VCAM-1 and E-selectin on endothelial cells exposed to angiogenic factors (193). Similar results were obtained by different angiogenesis inhibitors such as SU6668-Orantinib (194) and angiostatin (195), validating this approach. This class of drugs is being developed for tackling neoangiogenesis, a hallmark of cancer, and the effect on CAM expression could be exploited for targeting NMs for achieving synergistic tumor growth inhibition.

#### Combination of Factors

Upregulation of ICAM-1 in cervical cancer cell lines can be achieved by irradiation alone, however combining gamma irradiation with the pharmacological agent retinoic acid leads to an additive effect, superior to either agent alone (196). Possibly, this approach of testing different rational combinations of agents can yield the most efficient strategies for enhanced NM targeting.

## Limitations and Risks of Using CAMs as Targets for NMs

Although CAM-targeted NMs have shown promising therapeutic results in pre-clinical settings, there are challenges that hamper their clinical translation. Targeting success depends on several parameters, including relative expression of CAMs in diseased and normal tissues, binding affinity and avidity, and target trafficking and turnover. Moreover, the properties of the carrier molecule itself or ligand determine the stability, biodistribution, general toxicity, and safety of the vascular-targeted system, making it difficult to predict the feasibility and clinical efficacy of endothelial CAM-targeted NM formulations for the treatment of cancer or inflammatory disorders.

### a. Endothelial CAM Expression Levels

Since pathological factors modulate CAM expression in the disease site, it is important to pre-determine the target-CAM's expression level, both in the diseased tissue and in normal tissues, as this ratio may play a significant role in the success of drug delivery. In case the CAM is almost exclusively expressed on the inflamed endothelium at high density (i.e., E-selectin), a system with high binding affinity may give better outcomes. Moreover, if the target is expressed at low levels on vascular endothelium under physiological conditions, and upregulated in disease (e.g., ICAM-1) - a system with “marginal” avidity might be sufficient to anchor only to cells with pathological levels of the marker. Indeed, a controlled reduction of the ligand surface density on the carrier enhanced the selectivity of targeting to inflamed pulmonary vasculature in animal models, as determined by PET imaging (197, 198).

### b. Lack of Efficacy Due to Compensation

Targeting only one CAM alone may be insufficient in controlling disease as observed in a clinical trial of L-selectin neutralizing Ab (aselizumab), mentioned above. In these patients, the lack of efficacy was reported, even though very high level of L-selectin blockade on neutrophils was achieved (46). A possible explanation could be that inactivity of L-selectin was compensated by the synthesis of active E- and P-selectin ligands. The problem of CAM family member compensation can theoretically be circumvented with a pan-selectin antagonist. Bimosiamose (reported to prevent P-, E-, and L-selectin-mediated adhesion *in vitro*) has shown promise as potential therapeutic for human diseases, albeit the success was limited, and its development appears to be discontinued. Since bimosiamose functions primarily by blocking E-selectin-mediated adhesion, it probably did not fully block L- and P-selectin activity and the remaining selectins could still be sufficient to mediate adhesion. Alternatively, it's possible that the other CAMs (ICAM-1, VCAM-1) could compensate for selectin blockage. Thus, a Pan-selectin antagonist in combination with blocking other CAMs should be considered for effective therapies for the prevention of leukocyte infiltration in inflammatory conditions.

### c. Adverse Effects Arising From the Mechanism

Given that CAMs play an essential role in leukocyte traffic into inflamed tissues, interfering with CAM functions by blocking their mediated activity may pose a risk. Neutrophils play a crucial role in the resolution of inflammation and blocking their activity might delay the healing process (18, 199). A disturbed balance of the neutrophil pool might also increase the risk of infections, and change neutrophil levels in some organs other than the affected tissue. Indeed, in a Phase II trial of traumatized patients with aselizumab, the incidence of infections was higher in the treatment group (46).

A further concern with CAM blockade and infection rates was identified in a drug blocking a counterpart of CAMs. Efalizumab, an mAb against integrin α_L_β_2_ (LFA-1, ligand for ICAM-1), reached the market for psoriasis treatment. In consistence with the mechanism of action, this mAb induced a marked increase in peripheral WBC count, due to blocking their migration into tissues. Additionally, it precipitated T-cells hyporesponsivness, through an effect on α_4_β_1_ integrin, which correlated with reduced ability to produce an immune response (200). These finding raised a question of safety of similar drugs and their potential to promote infections. Indeed, clinical trials confirmed increased susceptibility to bacterial infections with efalizumab therapy. Longer treatments, unfortunately, revealed an association to a potentially life-threatening condition - progressive multifocal leukoencephalopathy (PML) caused by a reactivation of JC polyomavirus. This rare serious side effect led to the removal of efalizumab from the market. Similar issues have been risen with another mAb against α_4_β_1_ (VLA-4, ligand for VCAM-1) natalizumab. Soon after being approved for treatment of multiple sclerosis in 2004, it was withdrawn from the market due to cases of PML. Following further risk-benefit analysis, natalizumab was re-introduced under a special prescription program. Since then, this drug was approved for the treatment of Crohn's disease by FDA (not by EMA). Interestingly, another integrin-blocking mAb, Vedolizumab, which binds to α_4_β_7_ integrins did not show potential to induce PML (200).

Another concern regarding blocking CAMs can be predicted in tumor treatment. Blockade or attenuation of CAM-mediated activity can interfere with or decrease the metastatic spread of cancer, and this indeed prevented the seeding of circulating melanoma cells in the lungs of mice (86). Yet, the number of tumor-infiltrating cytotoxic lymphocytes (mainly CD3^+^CD8^+^ T lymphocytes), which negatively correlates with cancer severity, can also be decreased and this in turn may hamper the antitumor immune response (21, 201, 202). Irradiation and pharmacological intervention have been proposed as strategies to enhance CAM expression in tumors with low basal expression, leading to better drug accumulation. Yet, circulating cancer cells utilize CAMs to promote their own trafficking to the pre-metastatic niche and seeding in secondary organs. Thus, upregulation in CAM expression during irradiation might contribute to, rather than inhibit cancer progression, and this should be taken into account. This emphasizes the complexity and the need for precision in local irradiation, or the necessity for a personalized approach to get the best therapeutic outcome.

These examples illustrate the potential serious adverse effects of non-selective inhibition of blood leukocytes' interaction with endothelium and their activation.

The challenge for the future is to closely characterize roles of different CAMs in each pathology and further identify leukocyte subpopulations and their trafficking molecules that have a beneficial role. A CAM-targeted NM that accumulates preferentially in the inflamed tissue and reduces, but not completely blocks neutrophil infiltration might be beneficial, as demonstrated in the ability of P-Esbp to attenuate excessive inflammation in injured liver tissue (142). Such systems limit the blockade of CAMs only to the specific disease sites and could lower the risk of generalized adverse effect. This will help to prevent collateral effects (infections, tissue injury, or metastasis) and will leave protective leukocyte extravasation and immune surveillance unaffected.

### d. Binding Affinity and Avidity

Features of the carrier itself, such as size, ligand valence, density, and carrier geometry, determine the binding affinity of the system to CAMs. High ligand densities on the surface of NPs, liposomes or polymer could be beneficial regarding the increase of binding affinity for the vascular endothelium, however they might be difficult to achieve. An efficient system should also exhibit high specificity to the target tissue, and high serum stability, to demonstrate a clear *in vivo* benefit.

Several CAM-targeted NMs demonstrated enhanced therapeutic efficiency over non-targeted counterparts in pre-clinical settings but failed to demonstrate clinical advantage (83). One of the limitations of immunoliposomes is the fast clearance by the reticuloendothelial system, especially of liposomes heavily decorated with antibody (or Ab fragment), and as a result decreased accumulation the target tissue. Small high-affinity non-antibody ligands (small glycomimetics, short peptides or aptamers) are less expensive and should demonstrate favorable clinical benefit for some of these new NMs.

### e. Target Trafficking, Intracellular Fate and Toxicity

The structure of the nano-carrier and drug release features determine the sub-cellular fate, therapeutic activity, and duration of the effects of the cargoes. Following binding to CAMs, the system can be released from the target, remain surface bound, or be internalized, depending on the carrier properties. The binding of certain ligands to CAMs can cause receptor-mediated internalization. This is the desired outcome for targeting drugs to selected cells. In contrast, for blocking the interactions mediated by CAMs, or for imaging purposes, internalization is not desired. The selected carrier must be biocompatible, with minimal side effects and preferentially accumulate at its target site.

Comprehensive research needs to be done to optimize factors such as drug-release rates, internalization, subcellular fate in cell culture, as well as pharmacokinetics, biodistribution and toxicity in naive animals. Studies must be also designated to titrate the optimal dosage, time window of administration, and time intervals for repeated injections of the attached/encapsulated therapeutic molecules. All these issues apply to all new drugs or drug delivery systems, and thus, they are not unique to CAM-targeted NMs. Yet, they are of special importance and will determine the outcome of the study (as demonstrated by the effect on patients treated with rivipansel) (57).

## Future Perspectives

**Scheme 2:**
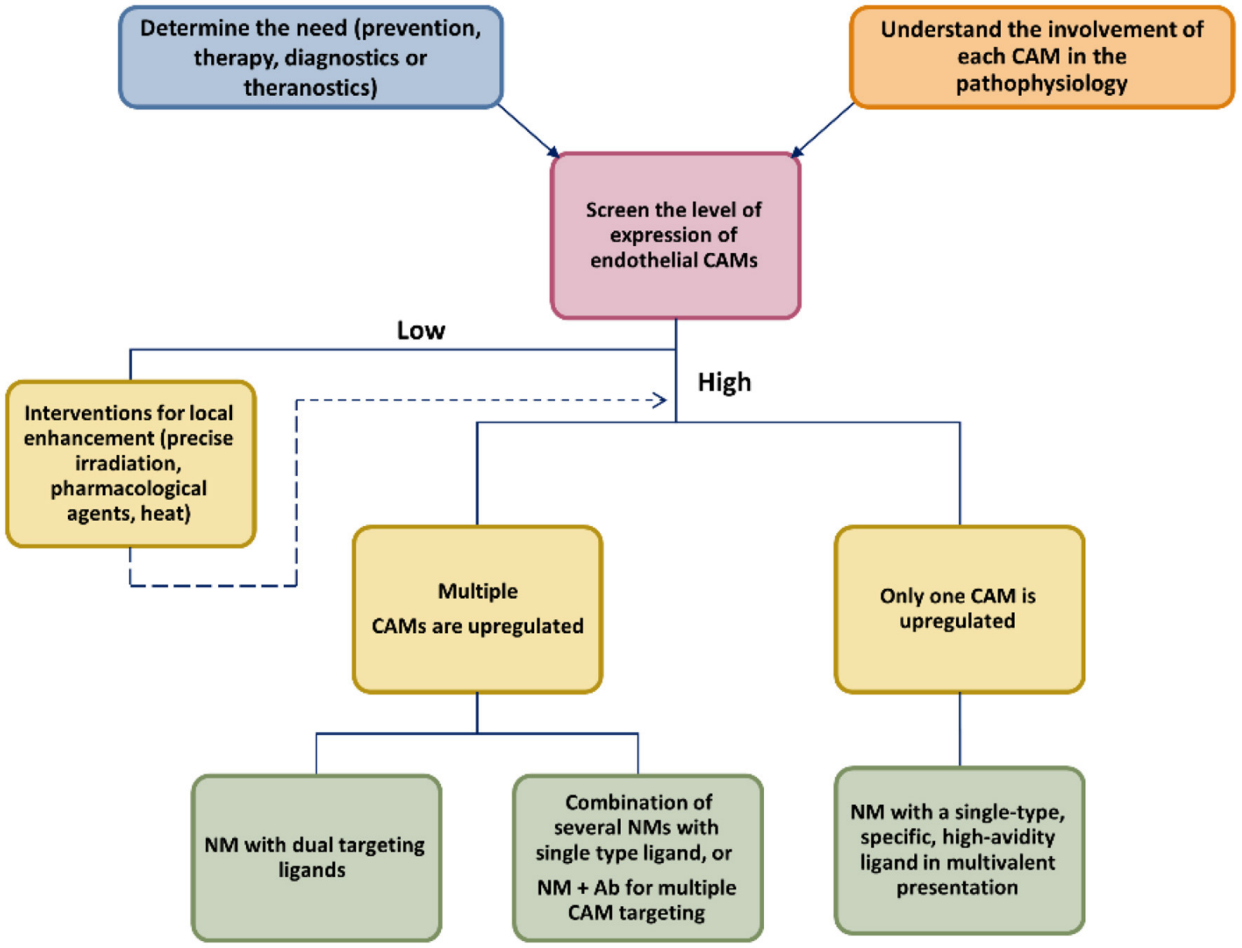
Approaching the development of new CAM-targeted NMs.

To date, a vast number of NM targeting CAMs has been developed and tested preclinically with various theranostic goals. No such system is close to regulatory approval, even though several non-NM drugs targeting CAMs have successfully found their therapeutic niche. We highlighted potential strategies that could help to maximize the potential advantages of NM and help clinical translation (Scheme 2).

A close reading of up-to-date literature signals us that future successful CAM-targeting NMs will be systems with a biocompatible carrier, have multivalent display of low Mw high-affinity ligands (peptide or aptamer), target two or several CAMs, with or without a conventional drug payload, depending on their therapeutic purpose. All of the critical elements (size of the carrier, type of the targeting ligand and ligand density, drug loading) should be investigated in order to find the optimal conditions to assure enhanced binding avidity, with minimal off-target effects, optimal PK properties, and avoid immune clearance. Furthermore, this NM could be combined with a pharmacological or physical agent that could potentiate the expression of the target CAM at the disease site, leading to improved targeting.

Choosing the relevant indication is another level of complexity. The roles of target CAMs should be well established and close attention should be paid to the possibility of compensatory up-regulation of other non-target CAMs as a result of successful targeting. We believe that NM translation should be directed first at diseases of those organs where NMs tend to accumulate (liver, kidneys), as this increases the chance of good clinical outcomes. Alternatively, CAM targeting could be combined with the local administration of NMs.

New inspiration comes from an array of “drug-free” macromolecular therapeutics for CAM targeting as an alternative to unsuccessful low-Mw drug inhibitors. These systems lack the presence of drug payload, which eliminates the potential for inducing drug-related adverse effects.

All of the building elements for CAM-targeted NM are well studied and an array of systems confirmed their preclinical efficacy. We are looking forward to seeing their clinical translation in the following years.

## Author Contributions

NM and MR were involved with drafting and writing the review manuscript, and including tables and figures. AD was involved in planning and writing this manuscript. All authors contributed to the article and approved the submitted version.

## Funding

The laboratory of AD is supported by Grant No. 1115/19 from the Israel Science Foundation (ISF), Grant No. 2017200 given by the United States–Israel Binational Science Foundation (BSF), by Grant No. 3-15008 from the Israeli Ministry of Health (MOH) and Grant No. 85698 from the Israeli Ministry of Science and Technology (MOS). MR is grateful to the Minerva fellowship for financial support.

## Conflict of Interest

The authors declare that the research was conducted in the absence of any commercial or financial relationships that could be construed as a potential conflict of interest.

## Publisher's Note

All claims expressed in this article are solely those of the authors and do not necessarily represent those of their affiliated organizations, or those of the publisher, the editors and the reviewers. Any product that may be evaluated in this article, or claim that may be made by its manufacturer, is not guaranteed or endorsed by the publisher.

## Abbreviations

ApoE^−/−^: apolipoprotein E knockout
BSA: bovine serum albumin
CAM: cell adhesion molecule
COPD: chronic obstructive pulmonary disease
CTs: clinical trials
CVS: cardiovascular disease
DDS: drug delivery system
DEX: dexamethasone
DOX: doxorubicin
Esbp: E-selectin binding peptide
ESTA-1: E-selectin thioaptamer 1
HPMA: N-(2-hydroxypropyl)methacrylamide
IBD: inflammatory bowel disease
ICAM-1: intracellular adhesion molecule 1
mAbs: monoclonal antibodies
MBs: microbubbles
MPIO: microparticles of iron oxide
MW: molecular weight
NM: nanomedicine
PAA: polyacrylamide
PAF: platelet-activating factor
PLGA: poly(lactic-co-glycolic acid)
PTX: paxlitaxel
Qa: quinic acid
RA: rheumatoid arthritis
RGD: arginine-glycine-aspartic acid
SCD: sickle cell disease
sLe^a^: sialyl Lewis^A^
sLe^x^: sialyl Lewis^X^
SPIONs: superparamagnetic iron oxide nanoparticles
TNFα: tumor necrosis factor alpha
USPIO: ultrasmall superparamagnetic iron oxide particles
VCAM-1: vascular cell adhesion molecule 1
VOC: vaso-occlusive crisis.
